# Coupling of a Major Allergen to the Surface of Immune Cells for Use in Prophylactic Cell Therapy for the Prevention of IgE-Mediated Allergy

**DOI:** 10.3390/cells13050446

**Published:** 2024-03-03

**Authors:** Konstantinos Mengrelis, Gerhard Niederacher, Lisa Prickler, Verena Kainz, Anna Marianne Weijler, Elisa Rudolph, Victoria Stanek, Julia Eckl-Dorna, Ulrike Baranyi, Andreas Spittler, Margarete Focke-Tejkl, Barbara Bohle, Rudolf Valenta, Christian Friedrich Wilhelm Becker, Thomas Wekerle, Birgit Linhart

**Affiliations:** 1 Department of General Surgery, Division of Transplantation, Medical University of Vienna, 1090 Vienna, Austria; mengrelis@gmail.com (K.M.); anna.weijler@meduniwien.ac.at (A.M.W.); elisa.rudolph@ewe.net (E.R.); 2 Institute of Biological Chemistry, Faculty of Chemistry, University of Vienna, 1090 Vienna, Austria; 3 Department of Otorhinolaryngology, Medical University of Vienna, 1090 Vienna, Austriajulia.eckl-dorna@meduniwien.ac.at (J.E.-D.); 4 Cardiac Surgery Research Laboratory, Department of Cardiac Surgery, Medical University of Vienna, 1090 Vienna, Austria; ulrike.baranyi@meduniwien.ac.at; 5 Department of Surgery, Division of Visceral Surgery and Core Facility Flow Cytometry, Medical University of Vienna, 1090 Vienna, Austria; andreas.spittler@meduniwien.ac.at; 6 Karl Landsteiner University of Health Sciences, 3500 Krems, Austria; 7 Department of Pathophysiology and Allergy Research, Center for Pathophysiology, Infectiology and Immunology, Medical University of Vienna, 1090 Vienna, Austria; 8 Institute of Immunology Federal Medical-Biological Agency (FMBA) of Russia, National Research Center (NRC), 115478 Moscow, Russia; 9 Laboratory of Immunopathology, Department of Clinical Immunology and Allergy, Sechenov First Moscow State Medical University, 119435 Moscow, Russia

**Keywords:** cell therapy, allergy prevention, Phl p 5, antigen cell labelling

## Abstract

Up to a third of the world’s population suffers from allergies, yet the effectiveness of available preventative measures remains, at large, poor. Consequently, the development of successful prophylactic strategies for the induction of tolerance against allergens is crucial. In proof-of-concept studies, our laboratory has previously shown that the transfer of autologous hematopoietic stem cells (HSC) or autologous B cells expressing a major grass pollen allergen, Phl p 5, induces robust tolerance in mice. However, eventual clinical translation would require safe allergen expression without the need for retroviral transduction. Therefore, we aimed to chemically couple Phl p 5 to the surface of leukocytes and tested their ability to induce tolerance. Phl p 5 was coupled by two separate techniques, either by 1-ethyl-3-(3-dimethylaminopropyl) carbodiimide (EDC) or by linkage via a lipophilic anchor, 1,2-distearoyl-sn-glycero-3-phosphoethanolamine-poly(ethylene glycol)-maleimide (DSPE-PEG-Mal). The effectiveness was assessed in fresh and cultured Phl p 5-coupled cells by flow cytometry, image cytometry, and immunofluorescence microscopy. Chemical coupling of Phl p 5 using EDC was robust but was followed by rapid apoptosis. DSPE-PEG-Mal-mediated linkage was also strong, but antigen levels declined due to antigen internalization. Cells coupled with Phl p 5 by either method were transferred into autologous mice. While administration of EDC-coupled splenocytes together with short course immunosuppression initially reduced Phl p 5-specific antibody levels to a moderate degree, both methods did not induce sustained tolerance towards Phl p 5 upon several subcutaneous immunizations with the allergen. Overall, our results demonstrate the successful chemical linkage of an allergen to leukocytes using two separate techniques, eliminating the risks of genetic modifications. More durable surface expression still needs to be achieved for use in prophylactic cell therapy protocols.

## 1. Introduction

Allergic disorders represent a persistent challenge to global public health [1]. Several strategies are currently available for therapy of established immunoglobulin E (IgE)-mediated allergies, such as pharmacotherapy and biologics, which neutralize IgE or inflammatory cytokines [2,3,4]. Allergen-specific immunotherapy (AIT) represents the only causative and disease-modifying treatment for allergic patients [5]. It comprises the subcutaneous [6] or sublingual [7] administration of natural allergen extracts and is associated with the induction of protective allergen-specific IgG and IgA antibodies, but also changes allergen-specific cellular responses of the innate and adaptive immune system [8]. The long-term clinical efficacy of AIT was confirmed in many clinical trials [9]. However, effective prophylactic treatment strategies for primary allergy prevention are still needed. In recent years, IgE sensitizations in early childhood have been analyzed using allergen microarrays, suggesting that allergic sensitization in children initially develops against a few molecules [10,11,12]. Since the corresponding allergen sequences are known, prophylactic approaches based on defined allergen molecules become feasible. In recent years, cell therapy approaches have emerged with the potential for targeted and sustainable interventions [13]. We aimed to develop a cell therapy strategy for achieving long-lasting prevention of IgE-mediated allergy by inducing robust allergen-specific tolerance.

In proof-of-concept studies, we have shown that irradiated BALB/c mice infused with autologous bone marrow cells (BM), that have either been transfected with a gene encoding the major grass pollen allergen, Phl p 5, or that have been retrieved from a transgenic mouse ubiquitously expressing Phl p 5, do not develop Phl p 5-specific IgE despite repetitive allergen immunizations [14,15,16]. The treatment also fully prevented Phl p 5-specific T cell responses and allergic airway inflammation, whereas immune responses towards the control allergen Bet v 1 remained unaffected. We subsequently developed an irradiation-free cell therapy protocol that also successfully prevents the occurrence of allergies in an allergen-specific fashion [15]. In these studies, we conditioned recipients only with a short course of mTOR inhibition and costimulation blockade. Moreover, we recently demonstrated that tolerance to Phl p 5 can also be induced by the transfer of Phl p 5-expressing CD19^+^ B cells instead of BM cells [17]. The robustness of this approach is underlined by the fact that Phl p 5 represents a highly immunogenic and allergenic molecule in allergic patients (11). Moreover, the allergen composition could be adapted to sensitization patterns detected early in childhood, e.g., by combining the relevant epitopes in one molecule [10,11,12].

However, for eventual translation into clinical practice, major protocol enhancements would be a prerequisite. Notably, retroviral transduction of donor cells needs to be avoided since its use is currently insufficiently safe for non-life-threatening diseases. Instead, peptides or proteins stably decorating the surface of cells could be used, which might be achieved with chemical coupling.

The crosslinker 1-ethyl-3-(3-dimethylaminopropyl) carbodiimide (EDC) has been commonly used to couple carboxyl groups to primary amines. The intravenous (iv) infusion of EDC-treated antigen (Ag)-coupled peripheral blood or splenic leukocytes has demonstrated immunomodulatory effects in rodent models of multiple sclerosis [18], type I diabetes [19] and transplant rejection [20,21]. In addition, EDC-coupling of peanut extract and ovalbumin was shown to alleviate allergic symptoms and decrease allergen-specific IgE production in a mouse model [22]. A phase 1 clinical trial has affirmed the safety of iv infusions of encephalitogenic peptides coupled to autologous leukocytes via EDC treatment [23]. Although the mechanism is not fully clear, EDC is known to elicit antigen-specific immunoregulatory responses, with both Treg and dendritic cells (DC) playing a central role [24].

Alternatively, lipophilic anchors can be used for coupling antigens to the cell surface. 1,2-distearoyl-sn-glycero-3-phosphoethanolamine-poly (ethylene glycol)-maleimide (DSPE-PEG-Mal) is one of the most commonly used reactive phospholipids for the conjugation of peptides or other ligands to plasma membranes, liposomes, or other lipid nanoparticles, facilitating otherwise challenging access to homogeneous, lipidated proteins [25]. In contrast to EDC-coupling, this strategy represents a more physiological approach, allowing a directed anchoring of the protein in the membrane and presumably better retaining the function of other molecules on the cell surface.

Herein, we investigated chemically linking a major allergen to the cell surface of immune cells for use in prophylactic cell therapy.

## 2. Materials and Methods

### 2.1. Mice

Female BALB/c mice were purchased from Charles River Laboratories (Sulzfeld, Germany) and kept at the Centre of Biomedical Research under specific pathogen-free (SPF) conditions. Mice were used between 6 and 12 weeks of age with a body weight of 20 +/− 2 g. As a control, a previously described (12) transgenic mouse ubiquitously expressing Phl p 5 and green fluorescent protein (GFP) on a BALB/c background (referred to as Phl p 5-tg) was used. Phl p 5-tg mice were bred at the animal facility of the Medical University of Vienna. The transgene was confirmed by PCR as described (15). GFP expression acted as a surrogate for membrane-bound Phl p 5, whose presence was additionally confirmed by flow cytometry using an anti-Phl p 5 primary antibody. All experiments were approved by the local review board of the Medical University of Vienna and approved by the Austrian Federal Ministry of Science, Research and Economy, GZ: BMWFW-66.009/2021-0,276.441) and were performed in accordance with national and international guidelines for laboratory animal care.

### 2.2. Generation of Recombinant Phl p 5 for EDC Coupling

The gene encoding the major grass pollen allergen Phl p 5a was codon-optimized for *Escherichia coli* (*E. coli*) expression, synthesized by ATG:biosynthetics (Merzhausen, Germany) and cloned into the expression plasmid pET17b (Novagen, Darmstadt, Germany) along with an N-terminal 8x histidine tag followed by a Tobacco Etch Virus (TEV) cleavage site (Supplementary Figure S1). The recombinant protein was expressed in *E. coli* strain BL21(DE3) (Agilent, Santa Clara, CA, USA) after the addition of 1 mM isopropyl-ß-thiogalactopyranoside for 4 h at 37 °C. The protein was purified from the soluble fraction on a nickel-nitriloacetic acid (Ni-NTA) Superflow Column (Qiagen, Venlo, Netherlands) according to the manufacturer’s instructions. The soluble protein was eluted using 250 mM imidazole and dialyzed against phosphate-buffered saline (PBS) overnight in 3 mL Slide-A-lyzer G3 Cassettes with a 3.5 K molecular weight cut-off (MWco) (Thermo Fisher, Waltham, MA, USA) at 4 °C and 50 rpm. The 8x histidine tag of the purified Phl p 5 was cleaved by overnight incubation with TEV protease (1:30 TEV protease:Phl p 5 molar ratio) and 2 mM dithiothreitol (DTT) at 4 °C and 50 rpm [26]. Endotoxins were removed using a Pierce High-Capacity Endotoxin Removal Spin Column (Thermo Fisher) and endotoxin-free water (Thermo Fisher) according to the manufacturer´s instructions. Post-removal endotoxin levels were measured on an Endosafe Portable Test System (Charles River, Lyon, France). At times, cycles of endotoxin removal and subsequent measurement had to be repeated up to three times until levels dropped below 1 endotoxin unit (EU)/mL. Phl p 5 concentration was determined by a Micro Bicinchoninic Acid (MicroBCA, Thermo Scientific) protein assay (Thermo Scientific, Waltham, MA, USA) using a Nunc-Immuno flat-bottom 96-well plate (Thermo Fisher). Phl p 5 purity was confirmed by a 12% sodium dodecyl sulfate-polyacrylamide gel electrophoresis (SDS-PAGE) followed by overnight Coomassie Brilliant Blue (Abcam, Cambridge, UK) staining. The molecular mass of Phl p 5 was confirmed by matrix-assisted laser desorption ionization–time of flight (MALDI-TOF) mass spectrometry (Bruker, Billerica, MA, USA).

### 2.3. Generation of Lipidated Phl p 5

Plasmid generation and expression conditions were the same as described before for recombinant Phl p 5 used in EDC coupling. The protein sequence differed in only 3 amino acids, including a cysteine, which were added for lipidation at the C-terminus (Supplementary Figure S1). The protein was purified from the soluble fraction by Ni-NTA purification using a gradient from 0–500 mM imidazole in PBS buffer at 4 °C. The fractions containing the protein of interest were pooled after SDS-PAGE analysis. TEV protease was added to the sample (1:20 ratio of TEV:Phl p 5-cysteine (Cys)), followed by dialysis overnight against PBS and 1 mM DTT at 4 °C using a dialysis tubing with a 6–8 kilodalton (kDa) MWco (Spectrum Chemical, New Brunswick, NJ, USA). The cleaved His tag was removed by preparative RP-HPLC at 60 °C on a C4 column using a gradient from 5–30% acetonitrile (can) in ddH_2_O/0.1% TriFluoroacetic acid (TFA) in 5 min, followed by 30–70% ACN in ddH_2_O/0.1%TFA in 30 min at a flowrate of 10mL/min. The fractions containing the purified Phl p 5-Cys were analyzed by electrospray ionization (ESI) mass spectroscopy (MS), pooled, and lyophilized.

For lipidation, the purified Phl p 5-Cys was dissolved in PBS buffer pH 7.2 containing 20 mM Tris(2-carboxyethyl)phosphine (TCEP) at ~2,6 mg/mL concentration. The crosslinker DSPE-PEG8-Mal (Broadpharm, San Diego, CA, USA) was dissolved in DMSO at ~2,8 mg/mL concentration using an ultrasonic bath. For a standard reaction, 9 mg Phl p 5-Cys in 3.5 mL buffer and 2.5 mg DSPE-PEG-Mal in 900 µL dimethylsulfoxide (DMSO) were combined, and the pH was adjusted to 7.2. The reaction was vigorously mixed for 1–2 hours at room temperature and monitored by liquid chromatography (LC)-MS. Upon complete conversion to the coupling product, the reaction mixture was diluted with 10 mL of a solution containing 6 M guanidine hydrochloride at pH 4.7. The resulting Phl p 5-DSPE, further referred to as lipidated Phl p 5, was filtered and purified by reversed-phase high-performance liquid chromatography (RP-HPLC) on a C4 column with a gradient from 5–30% ACN in ddH_2_O/0.1%TFA in 5 min followed by 30–95% ACN in ddH_2_O/0.1%TFA in 40 min at 60 °C and a flowrate of 10mL/min. The fractions containing the purified, lipidated Phl p 5 were analyzed by ESI MS, pooled, and lyophilized.

### 2.4. Recombinant Bet v 1

Recombinant Bet v 1.0101 was produced in BL21 (DE3) *Escherichia coli* cells and purified by hydrophobic interaction chromatography followed by anion exchange chromatography.

### 2.5. Circular Dichroism Analysis

The proteins’ secondary structure was determined on a spectropolarimeter (Jasco J-810, Tokyo, Japan) using a 1mm quartz cuvette. Spectra were recorded at room temperature at a scan speed of 50 nm/min and were corrected by subtracting the corresponding baseline spectrum, which was measured for water at identical conditions. Results are the means of three measurements and are expressed as molecular ellipticity.

### 2.6. Western Blots

Protein gels were blotted using the Trans-Blot SD semi-dry transfer apparatus (Bio-Rad Laboratories, Hercules, CA, USA). Membranes were washed three times with PBST/bovine serum albumin (BSA) for a total of 30 min at room temperature (RT). Membranes were incubated overnight with rabbit antiserum diluted 1:5000 at 4 °C with mild agitation. The next day, the solution was discarded, and the membranes were washed three times for a total of 30 min at RT and incubated with detection antibody for rabbit antisera diluted 1:5000 for 1 h at RT with mild agitation. Next, membranes were washed three times for a total of 30 min at RT, and 2 mL AP buffer was added for pH adjustment for 2 min at RT and mild agitation. Five milliliters nitro-blue tetrazolium and 5-bromo-4-chloro-3’-indolyphosphate (NBT/BCIP) substrate was added, followed by ddH_2_O to stop the color reaction.

### 2.7. Isolation of Splenocytes

Spleens were isolated from naïve BALB/c mice, and single-cell suspensions were prepared as described [14]. Suspensions were red blood cell (RBC)-lysed using a custom-made ammonium–chloride–potassium (ACK) buffer that consisted of 24.82 g NH_4_Cl, 3 g KHCO_3_, and 0.111 g ethylenediaminetetraacetic acid (EDTA) in 500 mL of sterile water (final pH 7.4). To lyse, a freshly meshed spleen was centrifuged at 300 g for 3 min in a 50 mL falcon tube. The pellet was resuspended in 2 mL of 5% fetal calf serum (FCS)/RPMI. Briefly, 0.5 mL of ACK buffer was added to 2.5 mL sterile water, and the final 3 mL solution was added to the spleen for a total of 5 mL for exactly 45 s. The falcon tube was filled up to 50 mL with 10%FCS/RPMI and centrifuged at 250 g for 10 min. After an additional wash, the suspension was filtered through a 70 μm cell strainer, and splenocytes were counted. Blood was lysed in a 15 mL falcon tube in 10 mL of sterile water for 20 s followed by the addition of 1 mL HANK medium and centrifugation at 250 g for 10 min. After careful supernatant aspiration, the pellet was resuspended in 1 mL PBS, and leukocytes were counted.

### 2.8. Lipidated Phl p 5 Coupling

Approximately 10^7^ RBC-lysed BALB/c splenocytes were resuspended in 0.5 mL PBS supplemented with 100 μg purified, endotoxin-free lipidated Phl p 5. Cells were incubated at 37 °C for 1 h at 50 rpm, followed by thorough washing to remove unbound rPhl p 5.

### 2.9. EDC Coupling

Approximately 10^7^ RBC-lysed BALB/c splenocytes were resuspended in 0.5 mL PBS supplemented with 6.32 mg dissolved ECD and 100 μg purified, endotoxin-free rPhl p 5. Cells were incubated at 4 °C for 1 h at 50 rpm followed by thorough washing to remove unbound rPhl p 5.

### 2.10. Splenocyte Culture

Duplicates of 10^6^ Phl p 5-coupled splenocytes were cultured in Greiner Cellstar 24-well plates (Sigma-Aldrich, Burlington, MA, USA) in RPMI 1640 medium (Biochrome, Cambridge, UK) supplemented with 10% FCS (Linaris, Frankfurt, Germany), PenStrep (100 U penicillin, 100 μg streptomycin/mL; Sigma), 10 mM HEPES (MP Biomedicals, Santa Ana, CA, USA), 1 mM sodium pyruvate (Sigma-Aldrich), 1x non-essential amino acids (Sigma-Aldrich), and 10 μM β-mercaptoethanol (Sigma-Aldrich). Cultures were incubated for 48 h at 37 °C and 5% CO_2._ The harvested cells were prepared for flow and image cytometry.

### 2.11. Flow Cytometry

Approximately 10^7^ splenocytes were stained with CD4 (PE-Cy7), CD8 (APC), B220 (APC-H7), CD3 (V450), and rabbit polyclonal anti-Phl p 5 IgG (15) for 20 min at 4 °C in the dark. After washing, donkey anti-rabbit IgG (PE) was added for 15 min at 4 °C. Biotinylated Annexin V was diluted in 200 μL Annexin V buffer and incubated for 15 min at RT. The cells were washed thoroughly twice and then stained with streptavidin (FITC) in 50 μL PBS for 10 min at 4 °C. After 2 washes, the pellet was resuspended in 200 μL PBS containing 1 μL 7AAD (PercP-Cy5.5), vortexed, and analyzed immediately with a Fortessa flow cytometer (BD). Phl p 5-specific polyclonal antibodies were purified from rabbit serum raised against rPhl p 5a on a HiTrap Protein G HP antibody purification column (Merck, Rahway, NJ, USA) according to the manufacturer’s instructions.

### 2.12. Image Cytometry

Cells were stained with anti-Phl p 5 (rabbit polyclonal), anti-rabbit IgG (PE), anti-CD4 (APC), anti-CD8 (FITC), anti-B220 (APC-Cy7), and DAPI and run at a low rate in a concentration of 5 × 10^5^/75 μL at 60X magnification using INSPIRE software version ISX on an ImageStream MkII image cytometer (Cytek Biosciences, Fremont, CA, USA). At least 10^4^ focused singlets were acquired per sample. Doublets were eliminated upon gating. Single-color controls were run to set compensation. Compensation and data analysis were performed using IDEAS software version 6.2 (Cytek Biosciences).

### 2.13. Immunofluorescene Microscopy

Approximately 7 × 10^5^ splenocytes were spun for 3 min at 72× g. Slides were fixed with 4% fresh neutral buffered formalin (NBF) at RT for 10 min. Slides were then rinsed in PBS and blocked with 10% goat serum in PBS at RT for 20 min. Briefly, 0.5 μg rabbit polyclonal anti-Phl p 5 was added to each slide, diluted in 100 μL 2%BSA/PBS, and incubated overnight at 4 °C in a humidified chamber. The next day, slides were washed 3 times in PBS, and 1:1000 goat anti-rabbit AF568 (Invitrogen, Waltham, MA, USA) was added in 2%BSA/PBS at RT for 1 h. Nuclei were counterstained with 1:1000 Draq5 at RT for 20 min and washed 3 times in PBS. Slides were rinsed with ddH20, mounted with ProLong Gold Antifade, and left overnight to dry. Slides were stored at 4 °C until visualization on a Nikon Ti Eclipse confocal microscope at 100× magnification.

### 2.14. Splenocyte Transfer

Splenocytes were isolated and prepared as described [17]. Aliquots of 10^7^ RBC-lysed and Phl p 5-coupled splenocytes were transplanted into the tail vein of naïve or preconditioned BALB/c recipients. Each preconditioned mouse was injected intraperitoneally (i.p.) with 50 mg/kg rapamycin (at d-1 for EDC experiments and d-2, 0, +2, +4 for lipidated experiments) and 1 mg hamster anti-mouse CD40L antibody (MR-1; Bio X Cell, Lebanon, NH, USA) at d0.

### 2.15. Immunisation of Mice

All groups of mice were immunized subcutaneously (sc) with 5 μg Phl p 5, as produced for EDC coupling, and 5 μg rBet v 1 adsorbed to aluminum hydroxide (Alu-Gel-S; Serva, Heidelberg, Germany) according to the manufacturer’s instructions. Mice were immunized and tail-bled at 4-week (w) intervals. Sera were stored at −20 °C until analysis.

### 2.16. Allergen-Specific ELISA

Allergen-specific ELISA was performed as described [17]. Plates were coated with 5 μg of either rPhl p 5 or rBet v 1. Sera were diluted 1:20 for IgE and 1:500 for IgG isotypes. Bound antibodies were detected with monoclonal rat anti-mouse IgE or IgG1 (BD) diluted 1:1000 in 0.5%BSA/0.05%Tween20/PBS. Goat anti-rat HRP-labeled antiserum was added at a 1:2000 dilution in 0.5%BSA/0.05%Tween20/PBS at 37 °C for 30 min and then at 4 °C for 30 min. Plates were visualized with 100 μL ABTS (258 mg citric acid, 275,2 mg Na2HPO_4_, 20 mg ABTS, 2 µL H_2_O_2_, 20 mL H_2_O) per well at RT using a Spark plate reader at 490 nm (TECAN, Männedorf, Switzerland).

### 2.17. Statistical Analysis

The statistical analyses were performed using GraphPad Prism v8.2 (GraphPad Software, La Jolla, CA, USA). Differences between mouse groups were calculated using the two-sided Mann–Whitney U test. P-values below 0.05 were considered statistically significant. Due to the small sample size, no statistical evaluation was performed for the preliminary experiment shown in Figure 4A. Results were presented as dot plots, with lines denoting the mean and whiskers denoting the standard error of the mean (SEM). All in vivo results, with the exception of Figure 4A, depict pooled data obtained from two independent experiments.

## 3. Results

### 3.1. Production of Two Variations of Phl p 5 for Coupling to the Surface of Immune Cells

**Figure 1:**
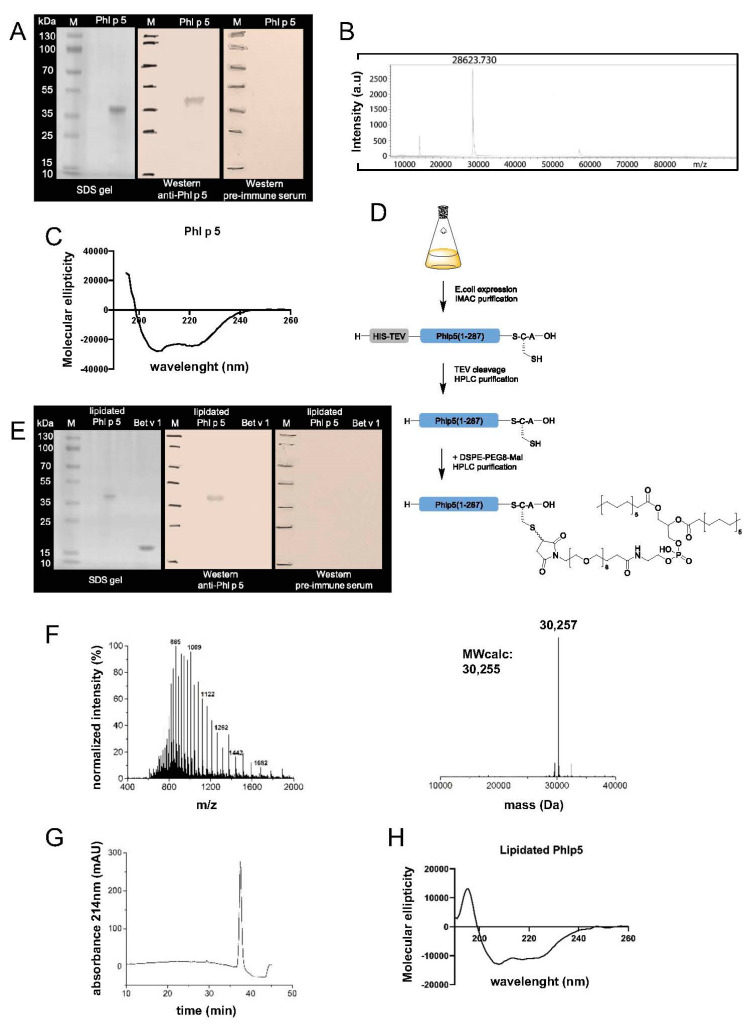
Biochemical analysis of recombinant and lipidated Phl p 5 allergens. A–C Analysis of Phl p 5 for EDC coupling. (**A**) SDS-PAGE (left side) and Western blot (right side) of purified Phl p 5, probed with a rabbit anti-Phl p 5 immune serum and the corresponding pre-immune serum as indicated. MWs (kDa) are shown on the left side. (**B**) MALDI-TOF analysis of Phl p 5. (**C**) Circular dichroism spectrum of Phl p 5. Molecular ellipticity (*y*-axis) is displayed for different wavelengths (*x*-axis). D–H analysis of lipidated Phl p 5. (**D**) Strategy for expression and lipidation of Phl p 5-Cys. (**E**) SDS-PAGE (left side) and Western blot (right side) of purified lipidated Phl p 5 and rBet v 1 as negative control, probed with a rabbit anti-Phl p 5 antiserum and the corresponding pre-immune serum. MW (Da) is depicted on the left side. (**F**) Mass spectrum of lipidated Phl p 5 (left) and deconvoluted spectrum (right). (**G**) Analytical HPLC chromatogram of purified Phl p 5. (**H**) Circular dichroism spectrum of lipidated Phl p 5 shown as in (**C**).

We selected a clinically important allergen, the major timothy grass pollen allergen Phl p 5a [11] for immobilization on the cell surface of immune cells. We investigated two distinct methods for coupling the allergen to the surface of BALB/c splenocytes. For this purpose, the cDNA encoding Phl p 5a (Genebank Accession No CAA52753) was inserted in the expression plasmid pET-17b along with an N-terminal 8x histidine tag and a TEV cleavage site (Supplementary Figure S1A). The protein was expressed in high yields as a water-soluble protein in *E. coli* and purified using Ni-NTA spin columns to homogeneity, as determined by SDS-PAGE. We confirmed the identity of the protein in a Western blot, probed with a rabbit anti-Phl p 5 antiserum and the corresponding pre-immune serum as a control (Figure 1A). The molecular weight was assessed by MALDI-TOF analysis (28,623.73 Da) and corresponded to the molecular weight calculated on the basis of the amino acid sequence (28,672.41 Da) (Figure 1B). The secondary structure of Phl p 5, which is known to consist of bundles of alpha-helices, was assessed by circular dichroism analysis, with minima at 209 and 222 nm, characteristic for an alpha-helical structure, indicating the correct folding of the protein (Figure 1C).

For the addition of a lipid linker, a cysteine residue was added at the C-terminus, as indicated in Supplementary Figure S1B. Phl p 5 used for lipidation was expressed with a short C-terminal tag comprising three amino acids (SCA) to enable chemoselective coupling of the PEG lipid (DSPE-PEG8-Mal) via the maleimide group to cysteine (Figure 1D). The cysteine-tagged Phl p 5 was purified from the soluble fraction of recombinant expression in E. coli by affinity chromatography. TEV cleavage was performed, followed by purification via RP-HPLC. The desired protein was obtained with high purity at a yield of 17 mg/L culture. Again, purity and identity of the expressed protein were confirmed by SDS-PAGE and Western blot probed with a rabbit anti-Phl p 5 antiserum, respectively (Figure 1E). MS analysis showed a molar mass of 28,936 Da, which corresponds well to the calculated mass of 28,934 Da. Lipidation was achieved by the reaction of the C-terminal cysteine with an excess of DSPE-PEG-Mal (MW 1,321 Da), which resulted in a highly pure product with a mass of 30,257 Da (calc: 30,255 Da) and a reaction yield of 60% (Figure 1F, G). As observed for Phl p 5 expressed for EDC coupling, lipidated Phl p 5 had a very similar secondary structure, indicating an alpha-helical fold of the protein, which was not essentially affected by the addition of DSPE-PEG-Mal (Figure 1H).

### 3.2. EDC-Mediated Phl p 5 Coupling Leads to Strong Phl p 5 Decoration on the Surface of Splenocytes Followed by Apoptosis

**Figure 2:**
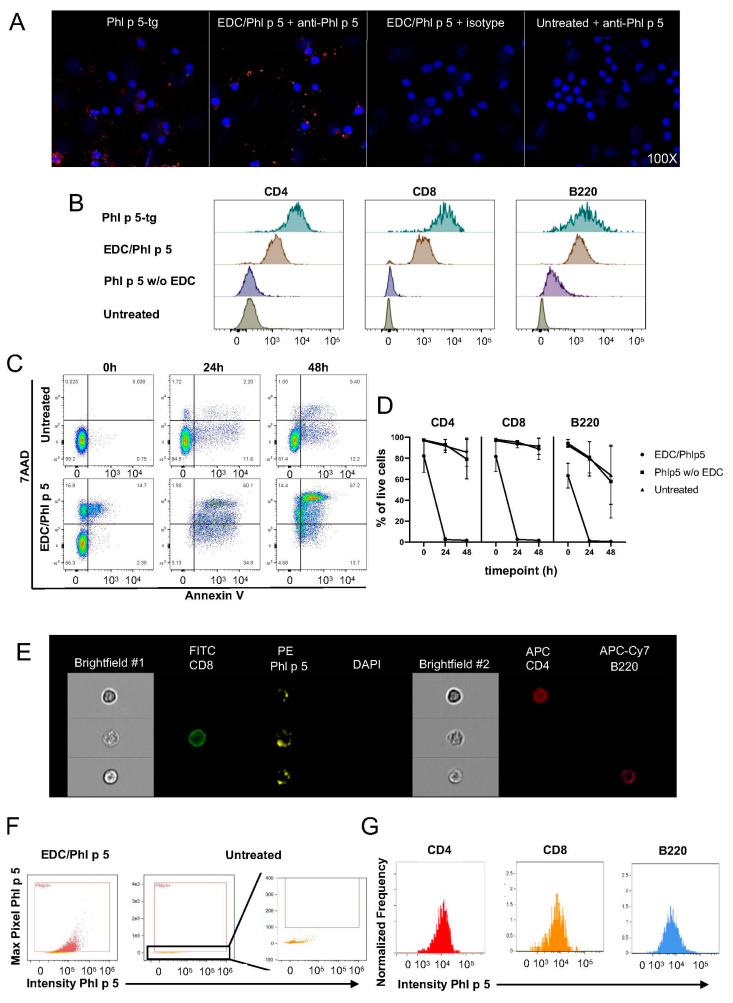
Analysis of EDC-mediated Phl p 5 coupling on murine splenocytes. (**A**) Cytospin immunofluorescent microscopic images of Phl p 5-tg, EDC-treated/Phl p 5-coupled (stained with rabbit polyclonal anti-Phl p 5 or isotype control) and naïve, untreated splenocytes (stained with rabbit polyclonal anti-Phl p 5). (**B**) Representative histograms show anti-Phl p 5 staining of Phl p 5-tg, EDC/Phl p 5, Phl p 5-treated (no EDC) as well as untreated splenocytes gated on CD4 (left), CD8 (center), and B220 (right). (**C**) Representative dot plots show 7AAD and Annexin V staining, gated on untreated (top row) and EDC-treated/Phl p 5-coupled (bottom row) splenic lymphocytes at 0 h (left), 24 h (center), and 48 h (right) in culture. (**D**) The plots show the percentage of live CD4, CD8, and B220 cells, respectively. Every point represents three independent experiments, and bars show mean +/− SEM. (**E**) Representative images of labelled EDC/Phl p 5 splenocytes showing fluorescence intensity and brightfield camera 1 and 2, generated using the ImageStream X MkII. At least 10^5^ cells were collected per setup. (**F**) Dot plots depict anti-Phl p 5 fluorescence intensity of live, focused, single EDC/Phl p 5 (left) and untreated (right) splenocytes. Zoomed box indicates the level of anti-Phl p 5 background stain and the positivity threshold. (**G**) Histograms show anti-Phl p 5 fluorescence intensity of CD4 (left), CD8 (center), and B220 (right) live, focused, single splenocytes.

One hundred micrograms of Phl p 5 was mixed with 10^7^ splenocytes in the presence of EDC for 1 h at 4 °C and mild agitation. Confocal microscopy of freshly prepared Phl p 5-coupled (hereafter referred to as EDC/Phl p 5) splenocytes stained with rabbit polyclonal anti-Phl p 5 antibody confirmed that the coupling was successful, as virtually all splenocytes stained positive for Phl p 5. In contrast, EDC/Phl p 5-coupled cells stained with a primary isotype control and fully stained uncoupled cells were negative, excluding false positive signals by antibody stainings, including the anti-Phl p 5 staining (Figure 2A,B). Next, we performed FACS analysis to assess whether EDC treatment links Phl p 5 to major splenic immune subsets with equal intensity (Figure 2B and Supplementary Figure S2). We found that over 99% of CD4+ T cells, CD8+ T cells, and B cells stained positive for Phl p 5, although the Phl p 5 MFI was lower compared to that of its Phl p 5-tg counterparts. Interestingly, Phl p 5-coupled B cells showed superior coupling efficiency compared to CD4+ and CD8+ T cells. In a negative control where splenocytes and Phl p 5 were mixed without EDC, we found that Phl p 5 was present on the surface of B cells at low levels but not on CD4 and CD8 cells. Next, we cultured allergen-coupled splenocytes for 48 h in complete RPMI (Figure 2C). Consistent with previous reports, EDC treatment triggered widespread cell death as indicated by Annexin V and 7AAD positivity, typical markers of apoptosis and necrosis, respectively. As dying or dead cells can stain false positive for Phl p 5, an accurate assessment of whether coupled Phl p 5 remains on the cell surface during culture was not possible. With regards to cell viability, B cells were found to become apoptotic upon EDC treatment more rapidly than CD4 and CD8 cells, as about 40% of them lost viability immediately after EDC treatment (Figure 2D). Incubation of splenocytes with Phl p 5 alone did not affect cell viability.

Next, we performed imaging cytometry on fresh EDC/Phl p 5 splenocytes (Figure 2E). Consistent with immunofluorescence and FACS data, rabbit polyclonal anti-Phl p 5 showed minimal background (Figure 2F). About 40% of splenic immune cells stained positive for DAPI and were excluded from downstream analysis, with the majority of those being B cells. Viable cells showed varying levels of Phl p 5 decoration, but overall B cells showed similar Phl p 5 MFI compared to CD4+ and CD8+ T cells (Figure 2G). Since no splenocytes survived 24 h post-EDC treatment, the experiment was not run with cultured cells. Overall, our data indicate that EDC treatment caused successful and strong Phl p 5 coupling in all immune subsets tested, followed by rapid and widespread cell death.

### 3.3. Addition of a Lipid Anchor to Phl p 5 Results in Strong Phl p 5 Decoration on Viable Splenocytes Followed by Antigen Internalization

**Figure 3:**
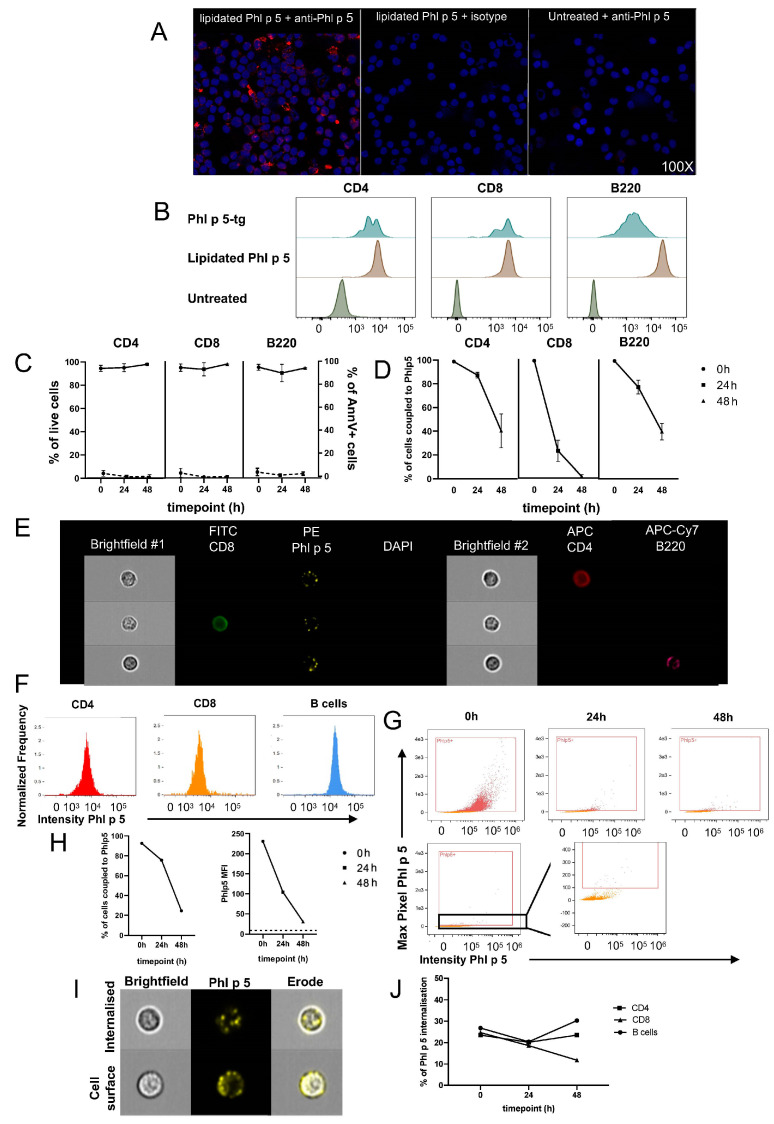
Analysis of lipidated Phl p 5 coupling on murine splenocytes. (**A**) Cytospin immunofluorescent microscopic images of splenocytes coupled to lipidated Phl p 5 (stained with rabbit polyclonal anti-Phl p 5 (left) or isotype control (center)) and naïve, untreated splenocytes. (**B**) Representative histograms show anti-Phl p 5 staining of Phl p 5-tg, lipidated Phl p 5-coupled as well as untreated splenocytes, gated on CD4 (left), CD8 (center), and B220 (right). (**C**) The plots show the percentage of live CD4, CD8, and B220 cells, respectively. Dashed lines depict the percentage of Annexin V+ counterparts. (**D**) The scatter plots show the percentage of live CD4, CD8, and B220 cells stained positive for cell surface lipidated Phl p 5. For (**C**,**D**), every point represents three independent experiments, and bars show mean +/− SEM. (**E–G**) Image cytometry analysis, representative one of two experiments. (**E**) Representative images of labelled lipidated Phl p 5-coupled splenocytes showing fluorescence intensity and brightfield camera 1 and 2, generated using the ImageStream X MkII. At least 10^5^ cells were collected per setup. (**F**) Histograms show anti-Phl p 5 fluorescence intensity of CD4 (left), CD8 (center), and B220 (right) live, focused, single, lipidated Phl p 5-coupled splenocytes. (**G**) Top row: Dot plots depict anti-Phl p 5 fluorescence intensity of live, focused, single lipidated Phl p 5-coupled splenocytes at 0 h (left), 24 h (center), and 48 h (right). Bottom row: Dot plots depict anti-Phl p 5 fluorescence intensity of live, focused, single untreated splenocytes. Zoomed box indicates the level of anti-Phl p 5 background stain and the positivity threshold. (**H**) show the proportion of live, focused, single splenocytes that stained positive for lipidated Phl p 5 (left) and the Phl p 5 MFI of the same population (right). (**I**) Representative images of labelled lipidated Phl p 5-coupled splenocytes showing mostly internalized Phl p 5 (top) and complete cell surface Phl p 5 staining (bottom) obtained using a mask denoting the internal portion of the cell, as defined by the erosion of 5 pixels into the brightfield cell perimeter. (**J**) shows the proportion of CD4, CD8, and B220 cells that showed signs of internalization at 0 h, 24 h, and 48 h post-coupling.

Next, we coupled the allergen to the splenocyte surface using Phl p 5 engineered with a lipid anchor. In order to couple the allergen on the cell surface, we mixed 100 μg lipidated Phl p 5 with 10^7^ splenocytes for 1 h at 37 °C and 50 rpm. The coupling efficiency and behavior of membrane-bound Phl p 5 were assessed analogously to the EDC coupling described earlier. Confocal microscopy revealed that coupling was successful (Figure 3A). FACS analysis demonstrated that Phl p 5 coupling was of similar intensity in CD4+ and CD8+ T cells, as seen in Phl p 5 transgenic cells, and an even higher intensity of Phl p 5 coupling in B cells compared to transgenic B cells (Figure 3B). Cultured lipidated Phl p 5-bound splenocytes showed almost complete survival, indicating that the addition of the lipid anchor does not mitigate viability (Figure 3C). Similar findings have been reported for other (artificial) membrane anchors [27]. This allowed us to examine the level of lipidated Phl p 5 remaining on the surface of viable allergen-coupled cells after 24 h and 48 h in culture (Figure 3D). CD8 cells lost about 80% lipidated Phl p 5 after 24 h in culture. In contrast, lipidated Phl p 5 was still present in over 80% of CD4 cells and 70% of B cells at 24 h. By 48 hours, ≈40% of CD4 cells and B cells remained positive for Phl p 5. Imaging cytometry confirmed that coupling was successful (Figure 3E). Of note, FACS and image cytometry data (Figure 3F) both indicated that B cells accumulated more Phl p 5 on their surface compared to CD4 and CD8 cells. Surface Phl p 5 was substantially reduced by 48 h in cultured cells (Figure 3G,H). The most plausible scenarios for the fate of splenocyte-bound Phl p 5 are either gradual internalization, dissociation from the cell surface, shedding, or a combination of these events. In fact, analysis of our data using an internalization wizard embedded in the INSPIRE data analysis software (Supplementary Figure S3) suggested that about 20% of all allergen-coupled splenocytes had recently internalized lipidated Phl p 5 at any given time (Figure 3I,J). Overall, our data indicate that the coupling efficiency of lipidated Phl p 5 was higher than that achieved by EDC using the same Phl p 5 concentration. However, allergen immobilization was transient due to internalization, with the majority of the lipidated Phl p 5 disappearing within 48 h.

### 3.4. Transplantation of Splenocytes Decorated via EDC with Phl p 5 Discretely Reduces Allergen-Specific IgG and IgE Levels

**Figure 4:**
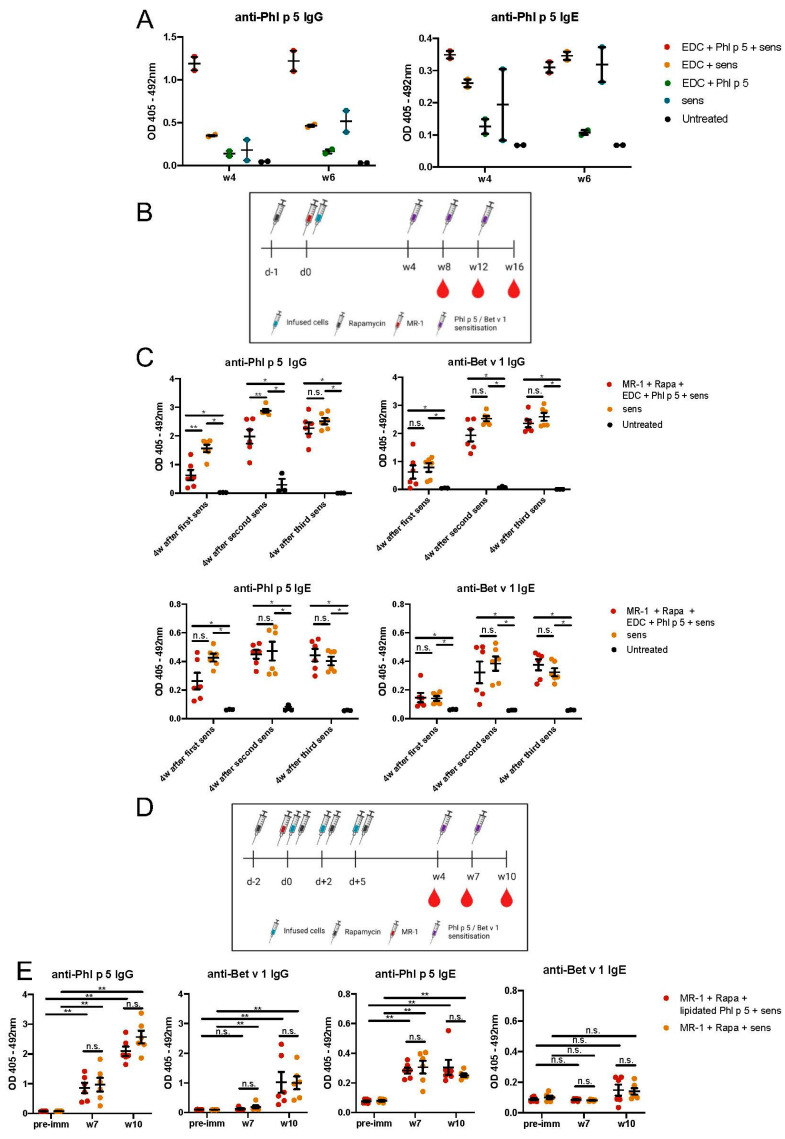
Transplantation of Phl p 5-bound splenocytes does not lead to specific tolerance. (**A**) Phl p 5-specific IgG (left) and IgE (right) serum levels of mice treated with 10^7^ EDC/Phl p 5-treated splenocytes (n = 2) at different time points. Results are presented as dot plots and the mean and SEM are shown. (**B**) Schematic representation of experimental setup with EDC/Phl p 5-treated splenocytes (n = 6), immunosuppression, and late sensitizations (four weeks post-cell transfer). (**C**) Phl p 5-specific IgG (top left) and IgE (bottom left) as well as anti-Bet v 1 IgG (top right) and IgE (bottom right) serum levels of mice treated with 10^7^ EDC/Phl p 5-treated splenocytes (n = 6) and rapamycin plus anti-CD40L (MR-1) at different time points. Results are presented as dot plots. The mean, SEM, and statistical significances are shown. (* *p* < 0.05, ** *p* < 0.01, n.s., not significant) (**D**) Schematic representation of experimental setup for repeated transfer of lipidated Phl p 5-treated splenocytes (n = 6), immunosuppression and late sensitizations (four weeks post-cell transfer). (**E**) Phl p 5-specific IgG (far left) and IgE (left) as well as anti-Bet v 1 IgG (right) and IgE (far right) serum levels of mice treated with 10^7^ lipidated Phl p 5-treated splenocytes and rapamycin plus MR-1 at different time points. Results are presented as dot plots. The mean, SEM, and statistical significances are shown. (* *p* < 0.05, ** *p* < 0.01, n.s., not significant).

Once we had established the successful Phl p 5 decoration and its in vitro dynamics, we evaluated the capacity of transplanted Phl p 5-coupled donor cells to induce tolerance. First, we injected 10^7^ EDC-treated, Phl p 5-coupled splenocytes into naive BALB/c mice (n = 2). Mice were immunized two weeks later with rPhl p 5 in alum, and blood was collected at w4 and 6. As immunological tolerance is characterized by a lack of antigen-specific antibody responses, Phl p 5- and Bet v 1-specific IgE and IgG responses were analyzed as primary outcome. Mice that received EDC-treated, Phl p 5- coupled splenocytes developed higher IgG and IgE serum titres against the allergen than sensitized mice that did not receive the cell product (Figure 4A). Another group of sensitized mice that received EDC-treated splenocytes (without Phl p 5, group: EDC+sens) showed allergen-specific IgG and IgE levels similar to those of sensitized mice that did not receive any cell infusion (group: sens). Infusion of the EDC/Phl p 5 cell product did not trigger a substantial humoral response without immunization (group: EDC+Phl p 5). Collectively, these data indicate that the transfer of EDC/Phl p 5-coupled cells primed a Phl p 5-specific response, triggering a strong humoral Phl p 5-specific immune response upon subsequent immunizations and not leading to tolerance or immunomodulation towards Phl p 5.

Next, we incorporated a short course of tolerogenic immunosuppression (the mTOR inhibitor rapamycin plus anti-CD40L (MR-1)), which previously allowed successful tolerance induction upon transfer of Phl p 5-transgenic bone marrow or B cells [15,17]. Mice (n = 6 per group) were subsequently immunized three times at 4 w intervals (either w1, 5, 9 (early sensitization; Supplementary Figure S4) or w8, 12, 16 (late sensitization; Figure 4B) with rPhl p 5 and rBet v 1 in alum. As previously observed [18], humoral responses were initially dampened due to the immunosuppressive preconditioning in early sensitized mice, i.e., 1 week after cell transfer, but increased with subsequent sensitizations occurring after immunosuppression clearance (Supplementary Figure S4). When the first sensitization of mice occurred 4 weeks after cell therapy, a slight reduction of Phl p 5-specific IgG and IgE responses was initially detected, which was significant for IgG and could not be observed for Bet v 1-specific antibody responses (Figure 4B,C). Eventually, high levels of anti-Phl p 5 and anti-Bet v 1 IgG and IgE antibodies developed 4 weeks after the 3rd immunization (Figure 4C). Thus, the transfer of EDC/Phl p 5-coupled cells does not downmodulate or tolerize the immune response towards Phl p 5 in a sustained manner, even when combined with pro-tolerogenic immunosuppression. This result is in alignment with the observation that no transferred cells could be detected in the peripheral blood 24 h after transfer (Supplementary Figure S5).

Finally, we performed a series of in vivo experiments transferring splenocytes chemically coupled using the lipidated Phl p 5 (Figure 4D) into autologous mice (n = 6 per group). Since we have elucidated that the presence of Phl p 5 on the cell surface is transient, we decided to perform three cell product infusions (10^7^ cells) at short intervals (d0, +2, +5). The time course of administration of immunosuppression and immunizations (w4, 7, 10) resembled that of a previously described allergy model of IgE tolerance using Phl p 5 transgenic cells [17]. Cell treatment failed to induce tolerance, as no reduction in anti-Phl p 5 levels was detected by ELISA compared to sensitized controls that did not receive the cell product (Figure 4E).

## 4. Discussion

In this study, we aimed to bring a previously described cell therapy approach closer to eventual clinical translation through linking an allergen to the cell surface with safer methods than retroviral transduction. The approach is based on the prophylactic application of allergen-expressing immune cells and is associated with the establishment of molecular chimerism in recipients for several months, and the development of robust and long-lasting allergen-specific tolerance. In previous work, we could show that very low levels of chimerism (so-called microchimerism) are sufficient for tolerance induction [15]. However, it is currently not known how long allergen expression needs to persist to achieve prevention from allergic sensitization. Here, we utilized two strategies for chemical linkage of a well-characterized and clinically important allergen, Phl p 5, to the cell surface of mouse splenocytes, including EDC-coupling and lipidated Phl p 5 modification with a lipophilic anchor. In relation to allergies, EDC has been used in two models of Th2 allergy responses: An ovalbumin-induced airway inflammation model and a whole peanut extract (WPE) food allergy model demonstrating significant antigen-specific serum IgE reduction [22]. Our in vitro results from EDC-coupling are consistent with the published data since we observed apoptosis in antigen-coupled cells, which has been described as a prerequisite to the induction of antigen-specific tolerance [24]. Unexpectedly and in contrast to reported protocols with other antigens, transplantation of autologous apoptotic EDC/Phl p 5 splenocytes did not prevent the Phl p 5-specific IgE and IgG response upon subsequent Phl p 5 sensitization, but indeed augmented the anti-Phl p 5 response. Consequently, we adapted the protocol by adding a short course of immunosuppression in accordance with our previously established protocols. However, except for a discrete initial reduction of Phl p 5-specific IgG and IgE responses, long-term tolerance could again not be induced. Distinct outcomes that were achieved in tolerance models using different allergens may highlight the role of the antigen itself for this approach. Phl p 5 specifically is known to be a highly immunogenic and potent allergen, eliciting strong immune responses in allergic patients. Moreover, the integrity of proteins might be differentially affected by the coupling procedure, influencing their in vivo efficacy. Furthermore, it has been suggested that Th1 responses are more successfully tolerated using EDC approaches [28].

Whereas the EDC tolerization mechanism presumably involves anergy-inducing direct antigen presentation in the absence of costimulatory signals, chimerism-based tolerance approaches heavily rely on clonal deletion and regulatory mechanisms for tolerance induction [29]. Adding a lipid anchor to a modified version of Phl p 5 enabled the straightforward chemical coupling of the allergen to the cell surface. The allergen was retained only for a limited time on the cell surface due to internalization, and we hypothesize that this period of time was not sufficient for tolerance induction. Internalized Phl p 5 is probably less potent in tolerization, as we observed in a previous study that cytoplasmic Phl p 5 led to less robust tolerance [16]. We attempted to circumvent this issue by repeatedly injecting coupled cells. However, the duration of decoration did not seem to suffice even under repetitive infusions, suggesting that the decoration of donor cells with the allergen needs to be strong, stable, and (relatively) long lasting.

The present study demonstrates that chemical coupling of an allergen for cell therapy is feasible, in particular with the use of a lipid anchor. However, current strategies do not suffice for robust tolerance induction in vivo, as previously demonstrated for allergen-expressing immune cells. Further studies are needed to understand the mechanisms underlying successful tolerance induction in our cell therapy approach. It is currently not known whether central or peripheral tolerance is involved or if regulation by cytokines plays a role [29]. Future studies will also need to focus on developing coupling strategies that lead to more stable immobilization of allergens on live immune cells.

## Data Availability

All research data are presented in the manuscript.

## Supplementary Materials

The following supporting information can be downloaded at: https://www.mdpi.com/article/10.3390/cells13050446/s1, Figure S1: Amino acid sequences of *E.coli*-expressed Phl p 5 used for EDC-coupling and lipidation.; Figure S2: FACS gating strategy; Figure S3: AMNIS gating strategy and internalisation masks; Figure S4: Schematic representation of experimental setup with EDC/Phl p 5-treated splenocytes, immunosuppression and early sensitisations (a week post cell transfer); Figure S5: Tracking adoptively transferred Phl p 5+ cells in the peripheral blood of BALB/c mice.
