# Supplementary material for: Coupling of a Major Allergen to the Surface of Immune Cells for Use in Prophylactic Cell Therapy for the Prevention of IgE-Mediated Allergy
Source: Cells. 2024 Mar 3;13(5):446. doi: 10.3390/cells13050446 (PMC10930660; doi:10.3390/cells13050446)

Supplementary Figure 1:
Amino acid sequences of *E.coli*-expressed Phl p 5 used for EDC-coupling and lipidation. N-terminal methionin, 8x histidine tag, a glycine linker followed by a TEV cleavage site are highlighted, as well as the C-terminal modification in Phl p 5 expressed for lipidation. An arrow indicates the TEV cleavage site. Molecular weights were estimated using the Swiss Bioinformatics Resource Portal Expasy.

(A) AA sequence of Phl p 5 used for EDC coupling

↓

MHHHHHHHHGGENLYFQSADLGYGPATPAAPAAGYTPATPAAPAEAAPAGKATTEEQKLIEKINAGFKAALAAAAGVQPAD

KYRTFVATFGAASNKAFAEGLSGEPKGAAESSSKAALTSKLDAAYKLAYKTAEGATPEAKYDAYVATLSEALRIIAGTLEVHAVKP

AAEEVKVIPAGELQVIEKVDAAFKVAATAANAAPANDKFTVFEAAFNDAIKASTGGAYESYKFIPALEAAVKQAYAATVATAPE

VKYTVFETALKKAITAMSEAQKAAKPAAAATATATAAVGAATGAATAATGGYKV

Number of amino acids: 288

Molecular weight (after TEV cleavage): 28672.41 Da

(B) AA sequence of mPhl p 5 used for lipidation

MHHHHHHHHGGENLYFQSADLGYGPATPAAPAAGYTPATPAAPAEAAPAGKATTEEQKLIEKINAGFKAALAAAAGVQPAD

KYRTFVATFGAASNKAFAEGLSGEPKGAAESSSKAALTSKLDAAYKLAYKTAEGATPEAKYDAYVATLSEALRIIAGTLEVHAVKP

AAEEVKVIPAGELQVIEKVDAAFKVAATAANAAPANDKFTVFEAAFNDAIKASTGGAYESYKFIPALEAAVKQAYAATVATAPE

VKYTVFETALKKAITAMSEAQKAAKPAAAATATATAAVGAATGAATAATGGYKVSCA

Number of amino acids: 291

Molecular weight (after TEV cleavage): 28933.71 Da

Supplementary Figure 2:
FACS gating strategy

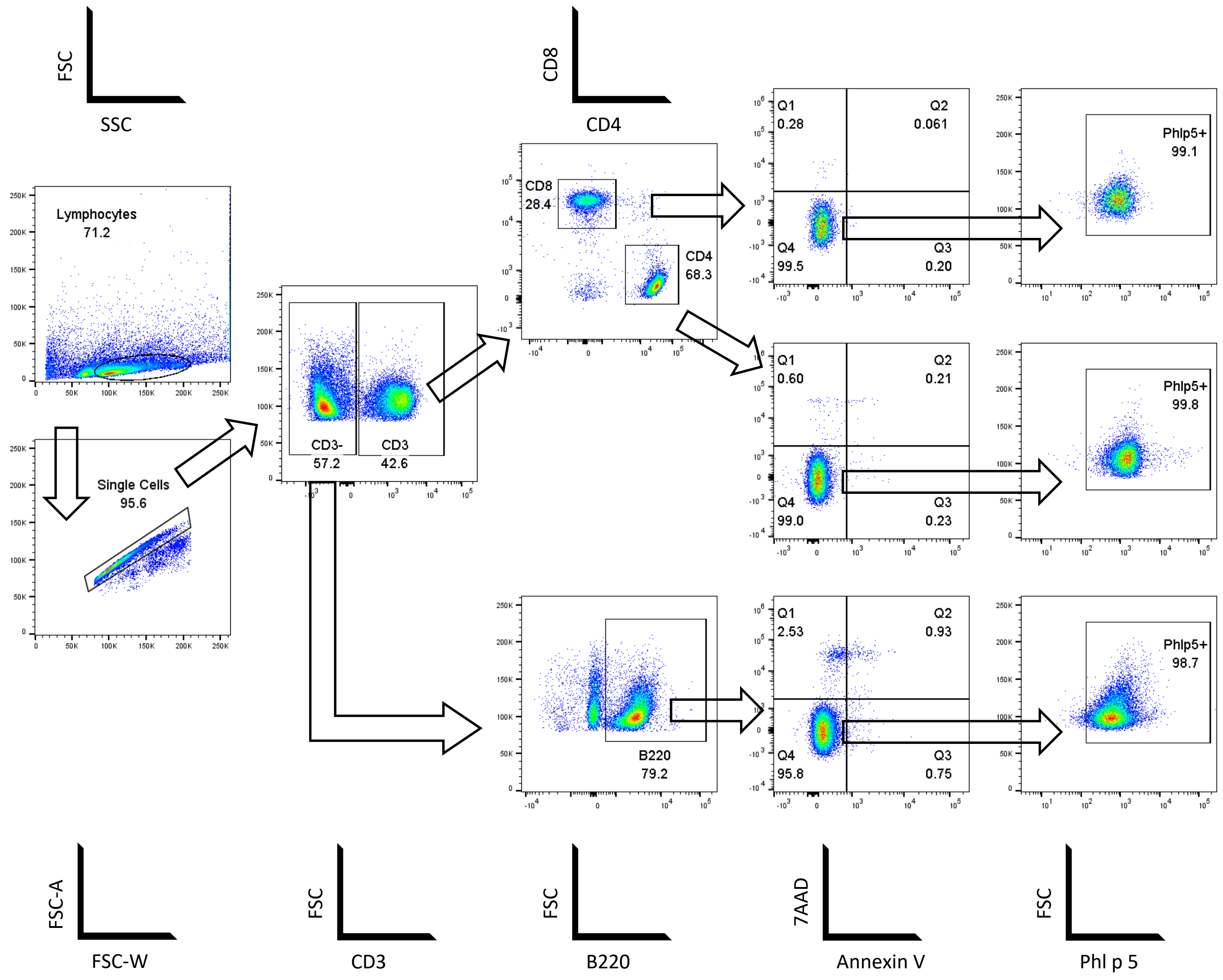

Supplementary Figure 3:
AMNIS gating strategy and internalisation masks

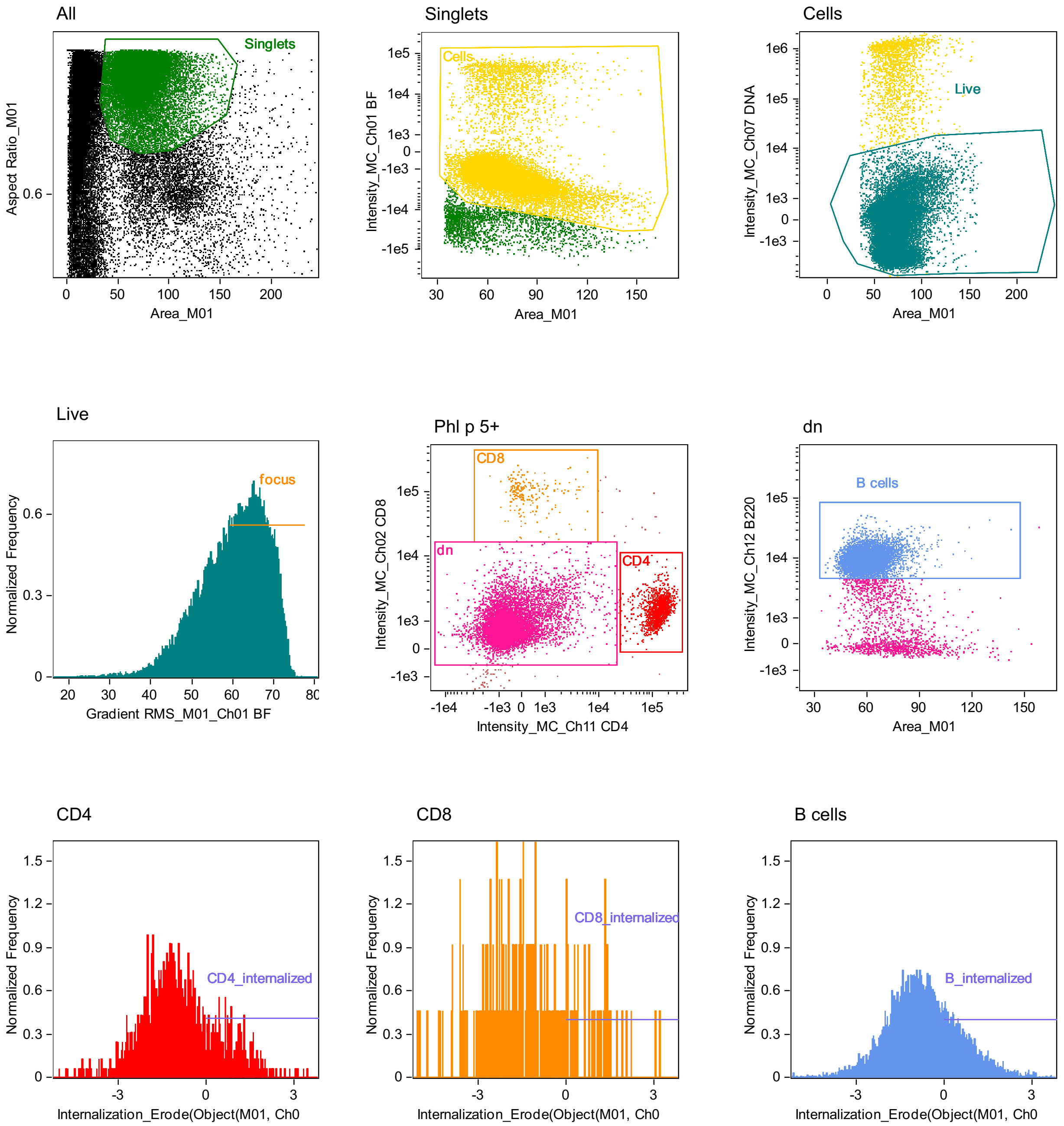

Supplementary Figure 4:
Schematic representation of experimental setup with EDC/Phl p 5-treated splenocytes, immunosuppression and early sensitisations (a week post cell transfer). (A) Schematic representation of experimental setup and (B) Phl p 5-specific IgG (top left) and IgE (bottom left) as well as anti-Bet v 1 IgG (top right) and IgE (bottom right) serum levels of mice (n=3) treated with 10^7 EDC/Phl p 5-treated splenocytes and rapamycin plus anti-CD40L (MR-1) at different time points.

A

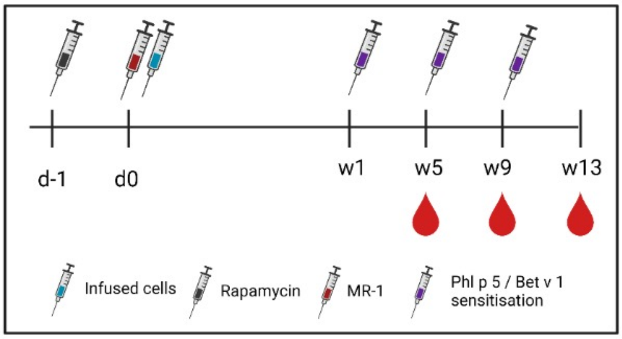

B

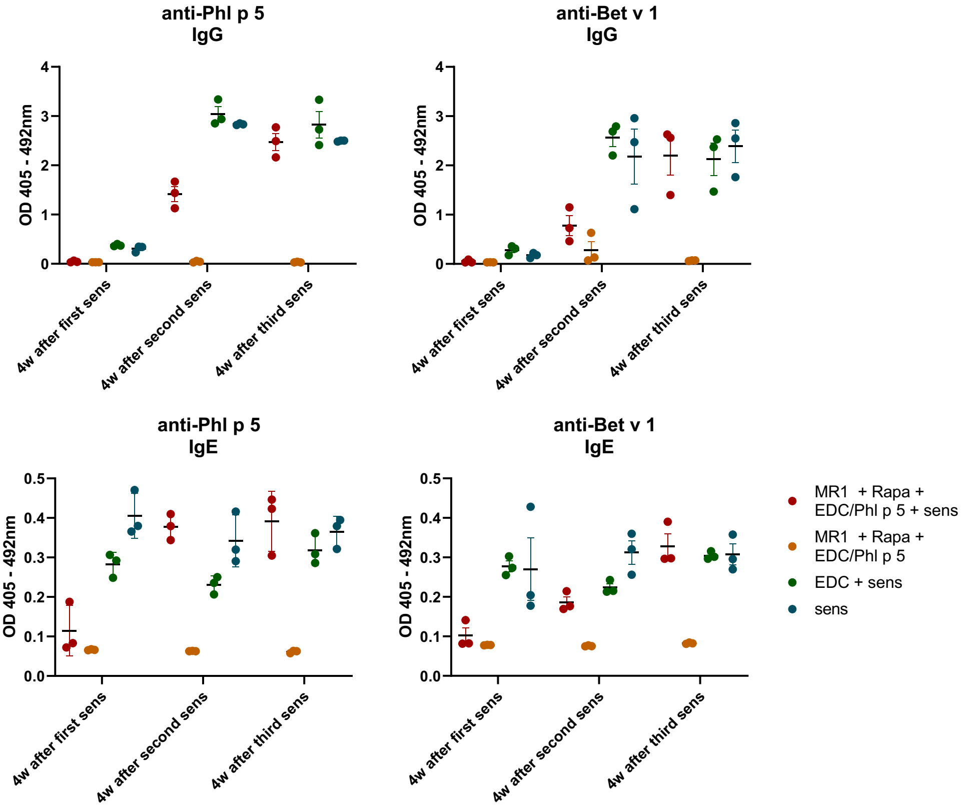

Supplementary Figure 5:
Tracking adoptively transferred Phl p 5+ cells in the peripheral blood of BALB/c mice before cell transfer (left panel) and 24h post cell transfer of EDC-coupled cells (right panel) by FACS

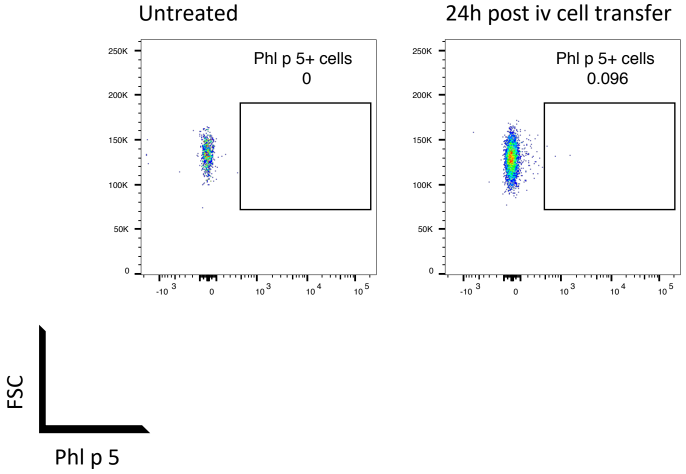

Supplement: Supplementary file 1 [file cells-13-00446-s001.zip › cells-2775691-supplementary.pdf]
